# The effect of Chinese vocational college students’ perception of feedback on online learning engagement: academic self-efficacy and test anxiety as mediating variables

**DOI:** 10.3389/fpsyg.2024.1326746

**Published:** 2024-06-24

**Authors:** Hui-Qin Cao, Cheon-woo Han

**Affiliations:** ^1^School of Primary Education, Hunan Vocational College for Nationalities, Hunan, China; ^2^Department of Education, Keimyung University, Daegu, South Korea

**Keywords:** the perception of feedback, academic self-efficacy, test anxiety, online learning engagement, SEM

## Abstract

Enhancing learning engagement is a critical challenge in online education. While previous research underscores the importance of feedback, recent studies have shifted focus to students’ perceptions of feedback, which significantly impact learning performance. However, empirical evidence on how these perceptions affect online learning outcomes is limited. Drawing on Self-Determination Theory, this study addresses this gap by employing SEM to analyze the relationships among feedback perception, academic self-efficacy, test anxiety, and online learning engagement. A total of 402 Chinese vocational college students (ages 18–19) completed questionnaires, with statistical analysis conducted using SPSS and Mplus. The study found that perception of feedback directly influences online learning engagement and indirectly affects it through academic self-efficacy and test anxiety, with a total effect value of 0.416. The findings offer valuable insights for educators and suggest directions for future research on feedback perception and online learning engagement.

## 1 Introduction

With the deep integration of the Internet and education, online learning has become a widely recognized and applied way of learning. But the reality of online learning for most students is less promising (Sun et al., 2023). Notably, due to the absent of face-to-face interaction and supervision, the lack of learning engagement has been identified as a critical concern in online environments (Kop et al., 2011). Especially for vocational college students who often possess weaker foundational knowledge and lower levels of non-cognitive skills, when they are not fully engaged, they tend to drop out of the learning process, and resulting in a low passing rate on final exams (Fred, 2012). Therefore, investigating the factors that can predict learning engagement and enhancing online learning performance of vocational college students have emerged as a critical challenge for educators in the field of online teaching.

Self-Determination Theory (SDT) developed by Deci and Ryan suggests that individuals’ behavior can be driven by three basic intrinsic motivators: autonomy, competence, and relatedness. When individuals perceive their actions as autonomous, feel competent in task completion, and establish positive relationships with others, they are more likely to exhibit positive behaviors and higher levels of engagement (Ryan et al., 2021). In this context, previous research on the determinants of online learning behavior has typically focused on intrinsic factors such as cognitive beliefs, intrinsic motivation, self-efficacy, and academic emotions (Corno and Anderman, 2016). Among which academic self-efficacy remains of great interest. Research has shown that learning resilience is influenced by self-efficacy (Hong et al., 2021). It predicts online learning engagement through the interaction of confidence and competence, and will in turn influence academic performance by regulating mood, effort and persistence (Ha and Han, 2022).

On the contrary, test anxiety may undermine the learners’ sense of competence. Students generally express tension in course examination and questioning their own abilities, which is related to the sense of ability needs according to the SDT (Majidifar and Oroji, 2015). When learners are anxious and nervous, they may doubt their abilities and may have negative expectations of their learning performance. In the online environment, teachers cannot ignore the negative impact, because students with test anxiety often have difficulty persisting with academic tasks or achieving good test scores (Bashir et al., 2019).

Researchers also generally believe that feedback, as a two-way communication process between teachers and students (Nicol, 2010), also has a great advantage in promoting learning engagement and online learning. A Recent systematic observations of UK classrooms found that students are more on-task with more experienced teachers who use more verbal behaviour including verbal feedback (Apter et al., 2020). Beaumont et al. (2011) expressed the feedback process as a dialogue loop. There are multiple opportunities for teachers and students to participate in the process, such as starting with teacher-student dialogue at task setting, through guidance as the task proceeds or feedback on performance during discussion, and through action planning. This kind of instant and in-depth interaction greatly promotes the support of online learning. And on the basis of SDT, students are more likely to remain engaged in a program when they feel connected and supported (Rutz and Ehrlich, 2016).

However, by placing the student at the center of feedback, the student’s role in perceiving, generating, making sense of, and acting on feedback is emphasized (Siekmann et al., 2023). The perspective of recent study shifted from the feedback to the perception of the feedback (Strijbos et al., 2021). Due to its prior position in the individual’s processing of feedback, the perception of feedback plays an important mediating effect, and how learners perceive feedback largely determines its impact on learning performance (Bolliger and Halupa, 2018). Some studies have found that perceptions of positive feedback can lead to stronger self-efficacy and stimulate positive individual learning emotions (Rowe, 2017). In a certain context, students with a more positive perception of teacher feedback had lower anxiety (Di Loreto and McDonough, 2014). Also, the perceived usefulness of feedback can give students a sense of control over future outcomes and inspire more effort or engagement in learning (Fong et al., 2016).

Although students’ test anxiety decreases online learning engagement (Bashir et al., 2019), students’ perceptions of feedback may promote online learning engagement by reducing test anxiety. Moreover, based on the negative effect of self-efficacy on test anxiety (Zhu, 2015), the perception of feedback might also influence online learning engagement through academic self-efficacy and test anxiety. Thus, exploring how feedback perception could be used to foster online learning engagement seems particularly relevant. However, there is a lack of empirical evidence demonstrating the impact of feedback perceptions on other variables related to online learning (Polat et al., 2022), especially there is not much quantitative research that illustrates the direct relationship between perceptions of feedback and learning engagement. This study addresses existing research gaps and uses structural equation modeling to investigate the effects of the perceptions of feedback, academic self-efficacy and test anxiety on online learning engagement and to analyze the structural relationships among these four factors.

## 2 Theory background and literature review

### 2.1 The perceptions of feedback

In the field of education, the perceptions of feedback (PF) are concerned with students’ perceptions of teacher’s feedback from the students’ perspective (De Kleijn et al., 2013). Previous research emphasized that PF is the first stage in individuals’ processing of feedback and only about how the learner perceives feedback, it was seen as an important interpreter and mediator for the relation between teacher activities and student outcomes (Mory, 2004).

With more in-depth research on feedback, more and more attention has been paid in the field of education to the interaction between internal and external feedback and students’ processing of the results of external feedback (Boud and Molloy, 2013). Narciss (2008, 2013) has developed the interactive tutoring feedback (ITF) model to promote evaluating tutoring feedback strategies for digital learning environments. ITF conceptualizes feedback in a digital environment as a multidimensional pedagogical activity, emphasizing two interacting feedback loops (the feedback loop of the learner and the feedback loop of an external feedback source). Based on the ITF model, Strijbos et al. (2021) proposed that PF should also be a multidimensional concept, focusing on how the recipients spontaneously experiences the external feedback content, or a whole feedback process, in terms of cognitive, meta cognitive, motivational, and affective responses. Furthermore, their study distinguished two broad dimensions: first, perceptions that relate to the cognitive function of feedback, such as in terms of perceived fairness, usefulness and acceptance; secondly, perceptions that relate to the motivational function of feedback (e.g. motivate the recipient’s willingness to improve).

Although identifying feedback characteristics related to students’ PF, such as its sources, content, quality, or efficiency (Hattie and Timperley, 2007; Berndt et al., 2018), is crucial (Wu and Schunn, 2020), many scholars concentrate on the relationship and influence between the PF and other academic factors. Especially, in an online education environment, Kim and Park (2018) emphasize the importance of PF for learning presence and online teaching. Teachers can facilitate learning participation and active interaction through perceivable near-real-time feedback that reflects the presence of the learner. This is critical for online learning.

### 2.2 Academic self-efficacy

Self-efficacy is an important component of social cognitive theory and related to academic tasks and performance is called academic self-efficacy, and can be understood as one’s confidence in achieving academic success (Hong et al., 2021). In the online leaning research field, students’ academic self-efficacy for completing a learning task using an online learning system is the most widely studied motivational construct and is considered as a key predictor of students’ online leaning performance (Pituch and Lee, 2006). Because many studies have shown a positive relationship between academic self-efficacy and academic performance (Han et al., 2017). Researchers found that increasing self-efficacy motivates learning helps learners regulate their emotions (Torres and Solberg, 2001; Wang and Lin, 2021), and chooses to persevere in the face of difficulty.

However, the current online learning self-efficacy of college students is moderate and the online environment and personal characteristics have a significant impact on it (Liu and Wen, 2021). Some interventions have achieved good results in prior study, such as success and failure experiences, attributions, verbal persuasion, and affective reactions, etc, (Bandura, 1989), but research on the intervention of verbal persuasion (eg. giving positive feedback) in teaching process has yet to be studied in depth.

### 2.3 Test anxiety

Pintrich and De Groot (1990) suggests that test-taking anxiety has two components: a worry component, which refers to students’ negative thoughts that can sabotage academic performance; and an emotional component refers to affective and physiological arousal aspects of anxiety. Li and Fu (2013) paid attention to e-learning anxiety and described it as the emotional state of tension and anxiety caused by various uncertain factors in the learning situation during the process of information processing in online learning.

Generally speaking, test anxiety impedes performance achievement. It was found negatively related to learning retention and task value (Watthanapas et al., 2023). Ma et al. (2022)’s study also found test anxiety levels were significantly related to psychiatric traits, students with greater mood swings were prone to greater test anxiety.

In China, vocational college students, especially freshmen, generally have test anxiety. A reason cannot be ignored is that most of them have experienced failure in college entrance exams, and their inner sense of loss and low self-esteem are more prominent (Li and Fu, 2013). In addition, the test anxiety in online learning is also related to online learning style. Due to the lack of face-to-face opportunities, students may suffer from emotional loss to some extent, such as loneliness, helplessness or lack of collective sense of belonging (Luo et al., 2008). And lack of network technology, be afraid of improper operation could lead to anxiety (Surjono, 2015). Therefore how to reduce test anxiety to promote online learning in vocational college still need to be strengthened.

### 2.4 Online learning engagement

Learning engagement as a variable representing the initiative and effort of students to participate in effective educational activities has long attracted the attention of the education community. A large number of literature point out that learning engagement is crucial to student’s success (Ye et al., 2023). Most importantly, learning engagement can improve a major issue with online courses: students’ feelings of isolation or is a key factor in solving students’ learning burnout (Dixson, 2010), and aslo have an influence on students’ satisfaction (Sulla et al., 2023).

Fredricks and Paris (2004) stated that student engagement should encompass behavioral, emotional, and cognitive engagement in the learning process. Dixson (2010) combined social constructive notions of learning and previous research findings, asserted that engagement is composed of consisting of attitudes, thoughts and behaviors, and communication with others, and therefore learning engagement is that students invest time, energy, thoughts, effort, and feelings into learning. Based on this perspective, Dixson (2010) developed one of the few scales that addresses engagement in online learning.

Since online learning activities are predominantly self-directed, researchers tend to explore strategies to enhance learning engagement from the perspective of motivation stimulation, especially internal motivation, which is more stable and lasting (Jung and Lee, 2018). There has been a great deal of relevant research on the inner factors, such as self-efficacy and perceived usefulness or students’ personality traits and stress perception (Quigley et al., 2022). Another factor that attract the attention of researcher is external feedback. Peer feedback was found to have a positive effect on learners’ social engagement, while instructor feedback could significantly influence the process and development of online discussions, which had a more important impact on engagement and learning outcomes (Junaidi et al., 2022). The results showed that the use of affective feedback did not significantly increase learning engagement, but the hybrid feedback strategy significantly increased their behavioral and cognitive engagement. Therefore, the impact of feedback on online learning engagement remains to be confirmed.

### 2.5 Research model and hypotheses

#### 2.5.1 Research hypotheses

This study proposed four hypotheses based on the theory and the related literature.

Some scholars believe that learning engagement is related to the PF. Van der Kleij and Lipnevich (2021) reported the way in which feedback is provided during the learning process will influence the PF and thus the level of student engagement in an online environment. Perceived accuracy of feedback is an important mediator. While perceived negative feedback leads to goal disengagement and sustained engagement to a lesser extent. Mayordomo et al. (2022) showed that providing feedback during the assignment affected higher levels of cognitive engagement. And meanwhile, the perceived valence of feedback was significantly associated with emotional engagement in online learning. Brown et al. (2016) investigated the PF can predicted self-regulated learning and academic performance. Although there is not enough empirical research to definitively elucidate the relationship between PF and online learning engagement, based on prior study, it could be inferred a hypothesis as follow.

H1: The perception of feedback directly and positively predicts the online learning engagement of vocational college students.

Existing research suggests that the relationship between PF and self-efficacy is reciprocal (Winstone et al., 2017). Particularly, when the PF is in a positive state, or when the belief of improvement based on feedback is displayed, the positive mood will be stimulated, and the students will better enjoy the learning process, enhance their learning confidence, and thus make more efforts. So, experiencing these emotion in the long run, the PF could also has a positive effect on self-efficacy. It has also been shown that even when feedback is perceived as having some negative components, it can produce positive activation emotions to a lesser extent and may influence students’ self-efficacy (Rowe, 2017). Especially when receiving constructive feedback, confident students may use feedback as a learning opportunity, in turn injecting feedback to improve their academic achievement and will spend more time reflecting on their perceived feedback (Fong et al., 2021).

However, self-efficacy could affect learning engagement through self-confidence and anticipation of one’s own ability. Domestic of china and foreign research both have show that academic self-efficacy could significantly positively predict online learning engagement (Galla et al., 2014). Students with high academic self-efficacy devote more time and energy to study, while students with low academic self-efficacy do not devote themselves fully in class and show apathy (Bashir et al., 2019). Combined with the above discussion, we can speculate that academic self-efficacy may have an intermediary effect on the PF and online learning engagement. The following research hypothesis was proposed.

H2: Academic self-efficacy mediates the relationships between the perception of feedback and online learning engagement of vocational college students.

A majority of research has explored test anxiety can negatively predict online learning engagement (Steinmayr et al., 2016). This means if students have a high level of test anxiety about online course learning, they may lose their attention to and participation in learning tasks, which will result in poor academic performance, and the reduced test anxiety will attract them to actively participate in teaching activities, also make them interact with teachers and classmates more easily and complete course tasks with greater interest (Polat et al., 2022). But excessive test anxiety triggers more mood swings in learners, especially in online learning environments, where face-to-face communication is lacking, the more stressful the test is, the more likely it is to produce a negative mindset, which in turn negatively affects learning engagement (Chapell et al., 2005).

Although there is not much evidence on the specific relationship between the perception of teachers’ feedback and students’ anxiety, Di Loreto and McDonough (2014)’s investigation still inspirational. The results of the data from the comprehensive writing exam questionnaire showed that students’ PF were significantly and negatively related to test anxiety (*r* = −0.52, *p* = 0.001), and those students who perceived teacher feedback more positively having lower levels of anxiety. So it can be inferred that PF helped to reduce their test anxiety. In particular, the academic performance of online courses is usually composed of test results, group discussions, personal presentations, and other parts. Encouraged by multidimensional feedback, students generally have more active participation in learning, which will correspondingly improve their regular grades and further reduce test anxiety. Thus, although test anxiety decreases online learning engagement, students’ PF may promote online learning engagement by reducing test anxiety, with the hypothesis as follows.

H3: Test anxiety mediates the relationships between the perception of feedback and online learning engagement of vocational college students.

It has been confirmed by numerous studies that academic self-efficacy has an impact on test anxiety (Zhu, 2015; Ma et al., 2022). Although individuals’ evaluations of the test and their own abilities remain the same, their belief in success helps them construct a buffer between the evaluation and anxiety, which therefore reduces negative emotions (Ha and Han, 2022). Moreover, previous studies have demonstrated that individuals with low academic self-concept are more likely to have test anxiety and that developing individual self-efficacy could effectively reduce test anxiety (Zhang and Wu, 2010; Zhang and An, 2016).

Test anxiety is not only an outcome variable of self-efficacy, but also a predictor variable of learning engagement (Liu et al., 2020). Online learning engagement is a combination of four dimensions: skill, emotion, engagement, and performance, and the stronger the students’ academic self-efficacy, the more they control and reduce the negative effects of anxiety on their academic performance (Sulla et al., 2023). And moreover, based on the negative effect of self-efficacy on test anxiety, the PF might also influence online learning engagement through academic self-efficacy and test anxiety. Accordingly, the following research hypothesis was proposed.

H4: Vocational college students’ perception of feedback indirectly affect online learning engagement through the chain mediating effects of academic self-efficacy and test anxiety.

#### 2.5.2 Research model

**FIGURE 1:**
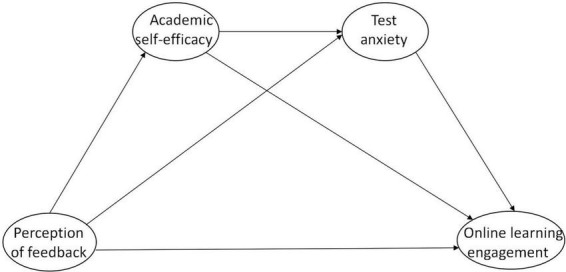
Hypothesized structural model between latent constructs.

SDT has been widely applied to understand how instructional practices, classroom environments, and teacher-student relationships can support students’ psychological needs and intrinsic motivation, ultimately fostering optimal learning experiences (Ryan et al., 2021). Based on SDT, this study synthesized and deduced four hypotheses above into a hypothetical research model to investigate the effects of the PF, academic self-efficacy and test anxiety on online learning engagement and to analyze the structural relationships among these four factors. The PF serves as the independent variable, while academic self-efficacy and test anxiety are mediator variables, online learning engagement will be the outcome variables. As illustrated in Figure 1.

## 3 Materials

### 3.1 Participants

Convenience sampling was adopted to recruit college students to participate voluntarily. As the only pilot province of the national smart education platform for vocational education in China, students from vocational colleges in Hunan Province were chosen as the participants. We chose two colleges, A and B, both with over 70 years of history and extensive teaching resources. These colleges collaborate, allowing students to cross-enroll in courses and receive mutual credit recognition.

As mentioned previously, the PF is greatly influenced by teachers and network technology platforms (such as experience, instructional design, instructional feedback language and feedback methods), in order to control measurement errors, it is suggested to select students of the same course with the same teacher. And according to Shumacker and Lomax (2016), the number of people participating in SEM studies should between 100 and 500 or more. After discussion and screening, 402 freshmen students (age 18–19, 44 male and 358 female) who took the Modern Chinese course in the college of elementary education were finally selected. Specifically, 252 of these 402 students were from college A, and the remaining 150 were from college B. They are taught by a with 12 years of teaching experience, use the same textbooks, online learning resources, online learning platform, and also the same course credits. Furthermore, when they entered the class, they were chosen at random by a computer and had no prior online learning experience on the learning platform.

### 3.2 Measures

#### 3.2.1 The online leaning engagement

The online student engagement scale developed by Dixson (2010) was adapted. This scale consisting of 19 items assess four types of online learning engagement: skills (i.e., Staying up on the readings), emotion (i.e., Really desiring to learn the material), participation (i.e., Posting in the discussion forum regularly), and performance (i.e., Getting a good grade). And it is a 5-point Likert scale ranging from 1 (not at all characteristic of me) to 5 (very characteristic of me).

#### 3.2.2 The perception of feedback

The present study adopted the feedback perception questionnaire (FPQ) compiled by Strijbos et al. (2010). The original FPQ contains 18 items on a 5-point Likert scale ranging from 1 (fully disagree) to 5 (fully agree). This questionnaire consists of five sub-scales. Four of these sub-scales have three items each: fairness (e.g., “I would consider the feedback I received in this course fair”), usefulness (e.g., “I would consider the feedback I received in this course helpful”), acceptance (e.g., “I would accept the feedback I received in this course”), willingness to improve (e.g., “I would be willing to improve my performance based on the feedback”) The fifth sub-scale, affect, is measured with six items: (e.g., “I would feel angry if I received this feedback”).

However, according to the FPQ developer advised in a paper (Strijbos et al., 2010), some students may not be able to distinguish between fairness, usefulness and acceptance, and in a sense, and they do conceptually belong to the cognitive function dimension of feedback, these three could combined into a new factor labeled PAF (perceived adequacy feedback). Moreover, the reliability of the new PAF has be confirmed higher internal consistency (Agricola et al., 2020). For these reasons, the PAF, Willingness to Improve (WI), and Affect (AF) scales of the FPQ were used in current study,

#### 3.2.3 Academic self-efficacy

The self-efficacy Scale produced by Pintrich and De Groot (1990) was used in this study to test the participants’ perception of academic self-efficacy. The scale consisted of 9 items regarding perceived competence (e.g., I expect to do very well in this class) and confidence in the class performance (e.g., I think I will receive a good grade in this class). The scale with 5-point Likert scale ranging from 1 (not at all true of me) to 5 (very true of me).

#### 3.2.4 Test anxiety

To assess the level of test anxiety, the test anxiety sub-scale from MSLQ (Pintrich and De Groot, 1990) was used. This sub-scale contains 5 items, of which 3 items refer to a worry or cognitive component, and two items refer to the emotional component. Responses were rated on a 5-point Likert s scale ranging from 1 (not at all true of me) to 5 (very true of me). Restricted words such as ‘in this course’ were added (eg., When I take a test in this course, I think about how poorly I am doing compared with other students).

#### 3.2.5 Reliability and validity of measurement

In this research, Cronbach’s α was conducted to determine the internal consistency of the questionnaire. As listed in Table 1, Cronbach’s α value of each measures ranged form 0.89 to 0.94. Even the Cronbach’s α value of three sub-scales of PF were above 0.7 (PAF: 0.94, willing to improve: 0.94, Affect: 0.93). And as for the sub-scales of online learning engagement, there were 0.89 (skill), 0.72 (emotion), 0.93 (participation) and 0.81 (performance). That’s to say, the questionnaire show an excellent internal consistency among the items (Hair et al., 2011).

**TABLE 1:** Construct reliability and validity of constructs.

Latent Variables	CR	AVE	FL	α	*t-*Value
Perception of Feedback	0.97	0.69	0.64–0.92	0.94	12.80–29.50
Academic Self-efficacy	0.92	0.54	0.63–0.80	0.90	12.68–16.94
Test Anxiety	0.89	0.62	0.69–0.91	0.89	13.18–17.62
Online Learning Engagement	0.95	0.57	0.53–0.90	0.89	9.14–19.31

FL, factor loading; CR, composite reliability; AVE, average variance extracted; α, Cronbach’s α.

The questionnaire was translated and modified from reliable instruments used in previous studies, and was evaluated by three educational experts and two professors of translation to ensure content validity. The external validity, convergent validity and discriminant validity are reported in detail as follows.

The external validity of the items was used to judge the extent of the study interpretation. A test was performed on the respondents in the first 27% and last 27%, if the t value (critical ratio) exceeds 3 (*** *p* < 0.001), the external validity can be considered significant. Table 2 shows that the *t* values range from 9.14 to 21.50 (*** *p* < 0.001), indicating that all items in this research were discriminating, and could differentiate the responses of different samples.

**TABLE 2:** Discriminant validity.

Latent Variables	1	2	3	4
1.Perception of Feedback	(0.83)			
2.Academic Self-efficacy	0.397	(0.76)		
3.Test Anxiety	0.478	0.362	(0.74)	
4.Online Learning Engagement	-0.490	-0.356	-0.446	(0.79)

The square roots of AVE values are in parentheses, and the other values are Pearson correlation coefficients.

Convergent validity was judged by FL, average variance extracted (AVE) and the composite reliability (CR). According to Hair et al. (2011), the FL and AVE value both should be greater than 0.50, and CR value higher than 0.70. In this study, the FL values of 51 items in the research ranged from 0.53 to 0.92, the AVE values of four latent variables ranged from 0.54 to 0.69, and CR value from 0.89 to 0.97, as shown in Table 2, indicate good convergent validity for each construct.

As the ideal discriminant validity, Hair et al. (2011) proposed that the AVE square root of each latent variable should be higher than the Pearson correlation coefficient of other latent variables. The results showed that the four latent variables had good discriminant validity in the present study, as shown in Table 2.

### 3.3 Data collection procedure

We distributed the questionnaire during the final exam review week in late January 2023, and provided a brief explanation of it. It was emphasized that the survey was anonymous, assured no teacher or school would have access to their personal data and their responses would not affect academic performance. The survey was administered by using the Xuexitong application, which notification function could automatically reminds students to check their questionnaires through text message and phone calls. And all procedures were approved by the college’s Institutional Review Board (IRB) before the survey began. By January 31, 2023, a total of 402 questionnaires have been collected. Nineteen questionnaires were excluded from the statistical analysis because these did not be given an adequate number of responses. Finally, 383 data were used for analysis.

### 3.4 Course overview and materials

The course being taught is Modern Chinese, a mandatory subject for elementary education majors in vocational colleges. What’s more, as an online course of the national smart education platform for vocational education in China, the Modern Chinese course has certified as a provincial high-quality online open course by the Hunan Provincial Department of Education in 2020. This course lasted 17 weeks last semester, with 16 weeks of online learning and a final online exam on the 17th week. Students registered on Chaoxing Xuexitong application and followed the teaching plan to study the course material, complete language analysis, group activities and tests, also participate in discussions posted online. These learning activities were sent to each student with a week’s notice. Teacher provided simultaneous study guides and question and answer tutorials.

Most feedback in online teaching aims to provide specific guidance, delineating both strengths and areas for improvement. It acknowledges progress, offers constructive suggestions, and provides opportunities for further development in various Chinese language skills or promote more learning engagement. For example, usually, the scores and errors of students’ homework or classroom exercises could automatically flagged by the learning platform. But the teacher provided more elaborated information based on the results. The feedback samples similar to the following: “Well done on completing the grammar exercises! Your understanding of the concepts is evident in your accurate definition of the nouns and the adjectives. One aspect to pay closer attention to is the use of verbs and prepositions. Reviewing the grammatical rules and examples for verbs and preposition usage will help you strengthen this area.”

During class activities or exercises, the teacher often provided immediate verbal feedback either individually or to the whole class. But after class, both text-based feedback and verbal feedback on the student’s performance could be given through the private messaging function of the learning platform.

### 3.5 Data analysis

Prior to conducting the main analyses examining the structural relationships, basic analyses were performed using SPSS v.27. At first, the normal distribution assumption was confirmed by assessing the average values of each measured variable. Furthermore, Pearson correlation analysis was conducted to examine the relationships between the variables. Moving forward, a structural equation model (SEM) analyses in Mplus v8.3 were used to examine the relationships among the constructs. The confirmatory factor analysis (CFA) was performed firstly to assess the fit of the measurement model and examine the structural relationships between latent variables by using the maximum likelihood (ML) estimation method. As a specific criterion, a good model fit was determined if χ^2^/*df* values were less than 3, the comparative fit index (CFI) and the Tucker-Lewis index (TLI) were 0.90 or higher, and the root-meansquare error of approximation (RMSEA) and the standardized root mean residual (SRMR) were less than 0.08; the factor loading (FL) values should also be greater than 0.50 (Hu and Bentler, 1999). Once the CFA model was finalized, the final step involved testing the hypothesized mediating model. We used bias-corrected bootstrapping analysis of re-extracting 5000 samples to verify the statistical significance of the multiple mediating effects (Preacher et al., 2007).

## 4 Results

### 4.1 Descriptive results

All variables were examined to ensure that they were normally distributed. Table 3 lists the descriptive results and zero-order correlations for all study variables. While the skewness and kurtosis absolute values in this study were both less than 2 (skewness 0.06- 0.89, kurtosis 0.07-1.33), which could ensure that they were normally distributed (Wu, 2018). The significant correlation coefficients among the measures were consistent with the theoretical foundations (e.g., the participants’ PF are positively related to their online leaning engagement, academic self-efficacy and test anxiety).

**TABLE 3:** Means, standard deviations, and correlations among all variables.

	*M*	*SD*	PAF	WI	AF	SK	EM	PA	PE	AS	TA
PAF	3.68	1.11	1								
WI	3.75	1.30	0.331**	1							
AF	3.25	0.99	0.611**	0.496**	1						
SK	3.28	1.01	0.269**	0.273**	0.282**	1					
EM	3.30	0.92	0.194**	0.119*	0.233**	0.296**	1				
PA	3.38	0.92	0.273**	0.242**	0.249**	0.365**	0.325**	1			
PE	3.35	0.91	0.161**	0.168**	0.163**	0.385**	0.255**	0.264**	1		
AS	3.49	0.86	0.395**	0.327**	0.448**	0.325**	0.185**	0.228**	0.328**	1	
TA	2.72	1.08	-0.423**	-0.0361**	-0.410**	-0.291**	-0.217**	-0.242**	-0.279**	-0.446**	1

PAF, Perceived Adequacy Feedback; WI, Willingness to Improve; AF, Affect; SK -Skill; EM - Emotion; PA - Participation; PE - Performance; AS - Academic Self-efficacy; TA - Test Anxiety.

*p < .05.

**p < .01.

### 4.2 Validation of measurement model

A confirmatory factor analysis was conducted to find out whether the measures reliably each latent factor according to the previously described steps.

**FIGURE 2:**
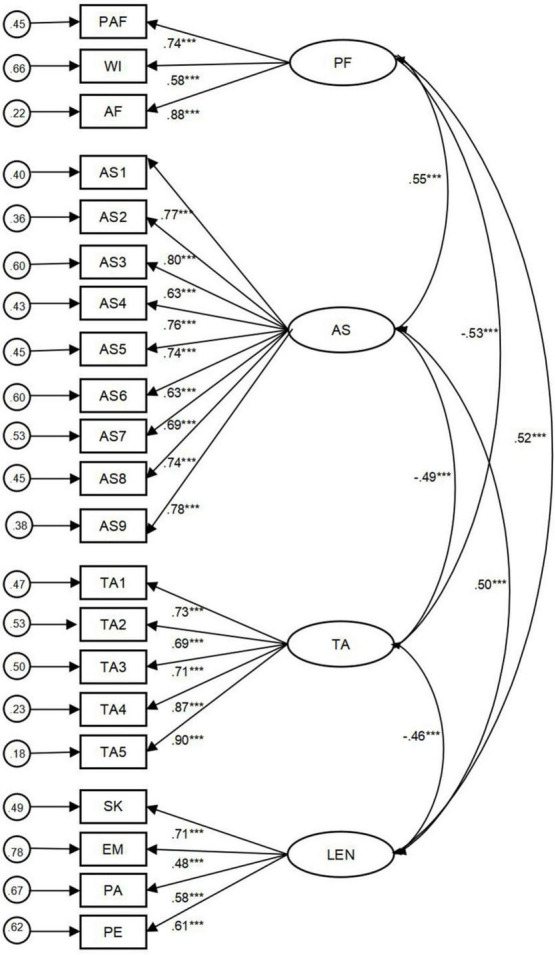
Final CFA Model With Standardized Coefficient. PF, Perception of Feedback; PAF, Perceived Adequacy Feedback; WI, Willingness to Improve; AF, Affect; AS, Academic Self-efficacy; TA, Test Anxiety; OLE, Online Learning Engagement; SK, Skill; EM, Emotion; PA, Participation; PE, Performance. *p < 0.05. **p < 0.01. ***p < 0.001.

The initial model consisted of the four latent constructs with their associated items mapped, the perception of feedback and online leaning engagement were identified through the second-order CFA, because the PF comprises three sub-constructs, and online leaning engagement has four. Results revealed good model fit (χ^2^/*df* = 1.50, RMSEA = 0.036. CFI = 0.953, TLI = 0.951, and SRMR = 0.051), see Table 2. All hypothesized FL were significant (*p* < 0.01 or *p* < 0.001), thus confirmed the major features of the hypothesized factor structure. The standardized FL and residual variances of the observed variables in the measurement CFA model are displayed in Figure 2.

### 4.3 Structural model analysis results

#### 4.3.1 Validation of Structural model

**FIGURE 3:**
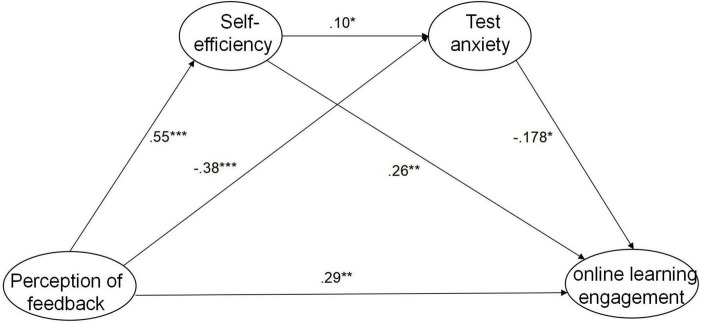
Latent Constructs of the Final Research Model With Standardized Regression Weights. *p < 0.05. **p < 0.01. ***p < 0. 001.

To test the hypothesized model that explained and predicted the relations between perception of feedback, academic self-efficacy, test anxiety and online learning engagement, an structural equation model (SEM) was created by adding the causal relationship to the measurement model presented in Figure 1 based on the theoretical background technology. The result was confirmed a good fit (χ/*df* = 1.51, RMSEA = 0.036, CFI = 0.953, TLI = 0.951, SRMR = 0.051, See Table 4), thus we chose it as the final research model. Figure 3 illustrates the associations among the latent constructs of the final research model with standardized regression weights.

**TABLE 4:** Summary of the model fit statistics.

Model	χ^2^	*Df*	χ^2^/*df*	RMSEA	SRMR	CFI	TFL
Measurement model	1824.369	1210	1.50	0.036	0.051	0.953	0.951
Structural model	1824.479	1211	1.51	0.036	0.051	0.953	0.951

#### 4.3.2 Path analysis

In regards to direct effect, the results determined that the PF positively affect online leaning engagement, the direct effect was 0.227 (β = 0.285, *p* < 0.01), which could verify hypothesis 1. And there were three significant indirect effects from the PF through academic self-efficacy and test anxiety to online leaning engagement. Specifically, the first one mediated through academic self-efficacy to online learning engagement, the standardized indirect regression weights were 0.114, the 95% confidence interval is from 0.041 to 0.216, which does not include 0 in the trust interval, this indicates our hypothesis 2 was verified. The second indirect effect from the perception of feedback through test anxiety to online learning engagement was 0.053, which does not include 0 in the trust interval (the lower limit is 0.01 and the upper limit is 0.119). Based on this, hypothesis 3 was verified. The third significant indirect effect from the perception of feedback to online learning engagement via academic self-efficacy and test anxiety was 0.022, the lower limit is 0.003 and the upper limit is 0.055, which also does not include 0 in the 95% confidence interval, thus the hypothesis 4 was confirmed. As it can be seen the total effect from PF to online learning engagement was 0.416, of which the total indirect effect was 0.189 (45.4%), as shown in Table 5.

**TABLE 5:** Indirect and indirect effects analysis.

Path	Standardized Coefficient (β)	95% Cl
PF → AS → OLE	0.114	[0.053, 0.198]
PF → TA → OLE	0.053	[0.018, 0.107]
PF → AS → TA → OLE	0.022	[0.006, 0.049]
PF → OLE (direct)	0.285**	
Total Effect	0.416	

**p < 0.01.

## 5 Discussion

In this study, the structural relationships among the perception of feedback, academic self-efficacy, test anxiety and online learning engagement are investigated. The main findings are as follows.

### 5.1 A direct relationship between the perception of feedback and online learning engagement

The PF had a direct positive impact on vocational college students’ online learning engagement. The results supported the hypothesis mentioned above that students’ PF during online learning determines how they engaged in learning. When students are aware that feedback is appropriate, they are more willing to make appropriate learning inputs based on the feedback. For example, the more fair and helpful feedback perceived during assignments and discussions, the more receptive the students are, thus influencing higher levels of cognitive change and stimulating learning inputs in skills, engagement, and performance. At the same time, as previous studies have shown, emotional engagement in online learning is significantly associated with the perceived valence of feedback (positive or negative) (Ali et al., 2018). The findings of this study illustrate that when vocational college students considered the feedback they received in learning activities as positive, they will show stronger positive emotions, and more confidence in participation or improvement, so as to make more efforts (Mayordomo et al., 2022). In addition, although it is a non-face-to-face learning environment, if the teacher has designed a variety of activities to guide learning, and students have truly perceived different feedback in this process, the learning presence would be enhanced and it is important for motivating satisfaction and participation with online learning (Kim and Park, 2018). Arguably, the PF could be an important psychological factor that helps students become aware of their emotions and regulate their learning behaviors during the online learning process.

### 5.2 The academic self-efficacy and test anxiety intermediary the role between the perception of feedback and online learning engagement

According to the indirect effects analysis results of the present study, some details are described below.

First of all, vocational college students’ PF has a positive impact on online learning engagement through academic self-efficacy. The PF can increase learning engagement by improving academic self-efficacy. Academic Self-efficacy plays an important role in students’ participation in learning. Students with high self-efficacy will develop a greater interest in academic activities by establishing demanding goals and taking action to achieve them (Rakoczy et al., 2013). In terms of behavior, cognition, and motivation, students with positive and relatively high self-efficacy beliefs are more likely to participate in classroom activities. However, as a motivational factor, students’ academic self-efficacy is inherently variable and is often affected by classroom situational characteristics (Linnenbrink and Pintrich, 2003), especially teachers’ feedback. For example, the study of Mayordomo et al. (2022) points out that when students hold a positive attitude towards feedback (not necessarily just positive feedback), they will show stronger positive activation emotion, thus being full of confidence in themselves and proud of being ready for improvement. In the long run, such positive emotional experiences can positively promote the improvement of college students’ academic self-efficacy. In particular, receiving accurate and adequate feedback that is helpful for academic improvement will give students more confidence in their course learning. Or receiving positive encouragement from instructor will make students more satisfied with course learning and further strengthen their belief in success, which will greatly improve their sense of self-efficacy. In this way, students can be motivated to put more effort and enthusiasm into online learning and achieve better academic performance.

Secondly, the vocational college students’ PF has an impact on online learning engagement through exam anxiety. The PF can increase learning engagement by reducing test anxiety. Test anxiety, as a negative academic emotion, has a negative impact on online learning engagement. However, receiving sufficient positive feedback that can stimulate the willingness to improve can relieve the anxiety of vocational college students to some extent, offset some insecurity and self-doubt in a non-face-to-face environment (Majidifar and Oroji, 2015), and shift students’ attention from the course examination to the learning itself. Stimulate interest in online course learning to promote online learning participation.

Thirdly, the PF indirectly affects the online learning input of vocational college students through the dual media of academic self-efficacy and test anxiety. During online learning, for example, students typically show greater academic self-efficacy when they feel that the feedback they got is fair, useful, and willing to accept, or perceive that it could stimulate positive feelings about their task performance and course learning or inspire beliefs about improvement based on feedback. This affirmation of self-ability can help students form a higher sense of academic self-efficacy (Dong et al., 2021). In addition, students with a strong sense of academic self-efficacy tend to be less affected by anxiety, and have a more rational view of course tests. Moreover, when facing more complex learning tasks, they could show greater learning motivation and actively regulate their emotions, and choose to persist in the face of difficulties. It can be said that Self-efficacy is attained through proactive emotional regulation and perseverance (Ha and Han, 2022) in order to promote learning engagement. From this perspective, it can also explain why self-efficacy has a great influence on learning engagement. As noted above, the PF has a static effect on online learning engagement through the media effects of academic self-efficacy and test anxiety.

### 5.3 Implication

The currents findings suggest the importance of PF emphasizing willing to improve, adequacy as well as positive affection to enhance their levels of learning engagement, This finding under the guidance of the SDT, highlight enhancing the opportunities or efficiency in students’ PF is crucial for creating an supportive online learning environment and fostering continuous engagement. Fong et al. (2021) suggested that targeting feedback appropriately to the learner’s needs and dispositions is central for positive PF and effective uptake. At first, as a facilitator, teachers should frame feedback as an opportunity for growth and improvement. For example, in group discussion activities, teachers’ feedback should be more aimed at stimulating learners’ internal feedback and providing feedback related to self-solving help. In addition, teacher prompt them to consider how they can apply the feedback to future learning activities and improve their performance.

Second, when delivering feedback, it is imperative to strive for maintaining interpersonal relationships and effectively conveying social emotions, thereby enhancing students’ positive PF. Correlation analysis of this study showed that the affect of PF has a relatively high correlation with the other variables. Therefore, teachers should endeavor to provide emotional guidance during the interaction process and assist students in recognizing and adjusting their emotions during the learning process (Mayordomo et al., 2022). An encouraging, compassionate, positive, and supportive tone should be used when providing feedback to promote a favorable perception of feedback among students (Van Popta et al., 2017). But it should also be used “moderately and cautiously” in teaching practice, especially when giving private text-based approval feedback, to avoid “complacency effect”. Because extra positive feedback may have made young student feel that they are good enough to achieve their academic goal without extra engagement (Sulla et al., 2018).

Third, actively carry out internal and external feedback loops in order to maximize students’ perceptions of feedback’s adequacy. Based on the interactive tutoring feedback mode (Narciss, 2013), online learning feedback must be effectively integrated into all elements of the design of all learning activities, including objectives, content, interaction, resources, and assessment. So teachers could provide timely and effective feedback to learners through the implementation of these activities, moreover should encourage students to participate in feedback interaction (Carless and Winstone, 2020).

It should be noted that one of the current study’s strengths is that it has confirmed that students’ academic self-efficacy and test anxiety successfully mediate the relationship between PF and online learning engagement. Based on the research result so far, various strategies that promote autonomy and mastery should be employed to fully stimulate students’ sense of self-efficacy in online teaching. And It is also an important goal for teachers to help alleviate students’ test anxiety in the context of online learning.

The core of online learning is the leading role of students in learning, while vocational college students often lack confidence in their learning ability due to the frustration of Gaokao (China’s college entrance examination) (Fu, 2021). Therefore, in the teaching process, teachers could design teaching activities with appropriate difficulty (i.e. challenging but not too difficult), and gradually enhance students’ sense of self-efficacy by supporting students to successfully complete teaching tasks. And it’s important for instructors and colleges to have a more diverse system for evaluating online courses in order to alleviate the stress that exams place on students. Furthermore, teachers should observe the benefits of their students from multiple perspectives and provide continuous interaction and immediate positive feedback in order to alleviate negative emotions and instill the belief that everyone can become a better person. This facilitates the elicitation of positive academic affect, accurate interpretation of instructional feedback, proper calibration of self-efficacy beliefs, reduce test anxiety, and allowing them to complete their online learning tasks with greater enthusiasm and effort (Brooks et al., 2019).

## 6 Conclusion and suggestions

### 6.1 Conclusion

The purpose of this study was to investigate how PF, academic efficacy, and test anxiety influence Chinese vocational college students’ online learning engagement and to determine the structural relationships among these four variables. To attain this research objective, a hypothesis research model was developed, and research hypotheses were formulated based on prior research. A questionnaire containing 51 items was disseminated to 402 Chinese freshman at vocational colleges in the province of Hunan, and 383 valid data were collected. The structural equation model analysis was carried out to verify the research hypothesis. Based on the research results and discussion, the conclusions of the study can be summarized as follows.

First, there is a direct relationship between the PF and online learning engagement. Second, it has confirmed that students’ academic self-efficacy and test anxiety successfully mediate the relationship between PF and online learning engagement. All three mediating paths were confirmed, including a single mediating effect of academic self-efficacy, a single mediating effect of test anxiety, and a third with the chain mediating effects of academic self-efficacy and test anxiety.

The findings indicate that PF is a significant factor in promoting engagement in online learning. Therefore, to boost vocational college students’ online learning engagement, it’s suggested to enhance the availability and effectiveness of PF, encourage academic self-efficacy, and alleviate test anxiety.

### 6.2 Limitations and suggestions for future research

Certain limitations in this study should be noted. Firstly, this study focuses on Chinese online learners in two vocational colleges, which may not fully reflect the reality of all Chinese online vocational colleges students. Therefore, the sample of this study could be further refined. We could continue to collect relevant data and carry out special or comparative studies across different ages, time spans, regions, or with different teachers.

Secondly, the cross-sectional design of the study limits generalizability to some extent. Therefore, in the following mediation model test, measures should be collected at different time points as variables are introduced. Additionally, to better operationalize and accurately measure online engagement, objective data collected by the online platform, such as login frequency and time spent on different stages, could be utilized. Furthermore, devices such as pupil change testers and eye movement meters (visual recorders) could be adopted to analyze various learning behaviors and obtain more accurate data regarding emotional engagement in online learning.

Thirdly, this study conducted quantitative research on the research topic but lacked qualitative research. In particular, the perception of feedback has complex influencing factors. Thus, further qualitative analysis should be conducted, involving a representative learning group to track the learning process, in order to establish a more objective and comprehensive relationship between variables.

## Data availability statement

The raw data supporting the conclusions of this article will be made available by the authors, without undue reservation.

## Ethics statement

The studies involving human participants were reviewed and approved by the Hunan Vocational College for Nationalities Ethics Committee. The studies were conducted in accordance with the local legislation and institutional requirements. Written informed consent to participate in this study was provided by the participants.

## Author contributions

H-QC: Data curation, Formal analysis, Funding acquisition, Investigation, Writing – original draft. C-wH: Methodology, Project administration, Supervision, Writing – review & editing.
